# General artificial intelligence for the diagnosis and treatment of cancer: the rise of foundation models

**DOI:** 10.1093/bjrai/ubaf015

**Published:** 2025-09-18

**Authors:** Ali A Tarhini, Palak Dave, Issam El Naqa

**Affiliations:** Department of Machine Learning, H. Lee Moffitt Cancer Center and Research Institute, Tampa, FL 33612, United States; Department of Machine Learning, H. Lee Moffitt Cancer Center and Research Institute, Tampa, FL 33612, United States; Department of Machine Learning, H. Lee Moffitt Cancer Center and Research Institute, Tampa, FL 33612, United States

**Keywords:** artificial intelligence, oncology, machine learning, generative AI, general AI, foundation models, AI agents

## Abstract

Artificial intelligence (AI) and its computer algorithms, including those of machine learning and deep learning, have been transforming the oncology field. Generative AI (Gen AI) and general AI technologies, which can be adapted to various tasks based on diverse inputs, are being increasingly utilized in healthcare applications. Foundation models, driven by Gen AI and general AI technologies, have enthralled the medical world, representing the next generation of large-scale AI tools in medicine that are trained on massive amounts of data and will make a major impact due to their versatility, high performance, personalization enhancement, and assistance of healthcare workers. That said, it is undeniable that there are multiple obstacles that these algorithms must overcome to be successfully utilized in the field. In this review, we first present a brief background and a history of the recent rise of Gen AI and foundation models. Next, we explore their applications in the diagnosis and treatment of cancer, specifically in radiology, pathology, precision medicine, personalization of care, and surgical oncology. Then, we discuss some of the limitations that could hinder general and Gen AI’s clinical translation.

## Introduction

**Figure 1.**
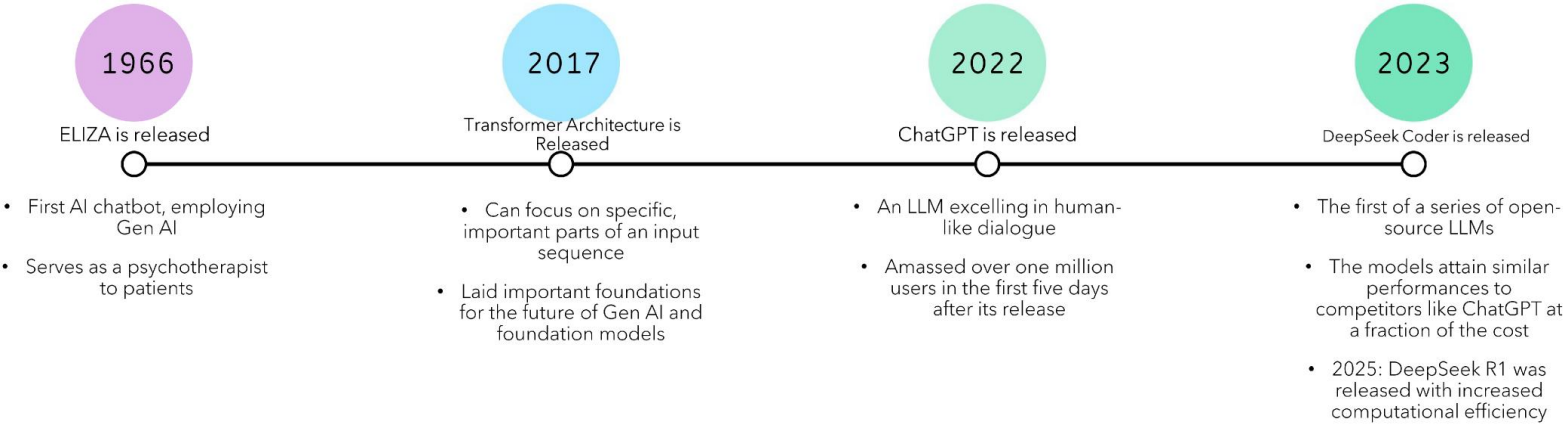
Timeline of the rise of generative AI and foundation models. Generative AI was in development as early as the 1960s but truly rose to the top of the medical field with the introduction of the transformer architecture. Later innovations, such as the release of ChatGPT and DeepSeek models, showed further promise for the potential routine application of generative AI and foundation models.^2^^,^^3^^,^^5^^,^^6^ Abbreviation: AI = artificial intelligence.

The early development of artificial intelligence (AI) was largely defined by predictive AI, such as traditional machine learning (ML) algorithms, utilizing extracted features from existing data to make predictions.^1^ Generative AI (Gen AI), a type of AI technology that can create another text or even images, art, and more from given descriptive text or other pieces of information, became a topic of discussion in the late 1960s, with the creation of the first AI chatbot, ELIZA.^2^ ELIZA served as a psychotherapist to patients, having the ability to match patterns from provided inputs to choose between predetermined responses.^2^ Importantly, predictive AI and Gen AI differ in the sense that predictive AI makes inferences and predictions from existing data, whereas Gen AI is able to generate new content from provided inputs.^1^ The evolution of predictive AI into Gen AI marks a major milestone in AI-involved cancer research. Gen AI continued to develop in the late 20th and early 21st centuries, but truly made its significant breakthrough in the late 2010s. A major milestone in the rise of Gen AI occurred in 2017, when Vaswani et al at Google released the first transformer architecture, an AI model based on neural networks, which can process and generate text, transforming input sequences into outputs.^3^ The model utilizes a sequence encoding and an attention mechanism that analyse the relationships between words in a sentence and allow the model to specifically focus on certain parts of an input sequence, comprehending context and circumstances better than previous models before it, and leading to higher performance.^3^ Transformers laid important groundwork also for the subsequent rise of foundation models, which are large-scale models trained on vast amounts of heterogeneous data.^4^ On November 30, 2022, ChatGPT was released by OpenAI as a “research preview.”^4^ This technology is a large language model (LLM), a foundation model that focuses on generating and processing language and specializes in natural language processing (NLP) capabilities^5^; the model excelled in human-like dialogue and spread immensely, amassing over 1 million users in the first 5 days of its release.^5^ Soon after, in November 2023, DeepSeek released its first of a series of LLMs, starting with DeepSeek Coder; recently, on January 20, 2025, DeepSeek-R1 was released, displaying high-level reasoning abilities and computational efficiency.^6^ DeepSeek models are open source, increasing the model’s visibility and interpretability. Additionally, these models achieve similar performance to commercial competitors such as ChatGPT at a fraction of the cost, with chip requirements and parameter quantities reduced during training.^6^ A timeline depicting the rapid rise and spread of Gen AI and foundation models is shown in Figure 1.^2–4^^,^^6^

Many of today’s AI algorithms are supervised models, which are created using known labels. These labels often need rigorous work and expert evaluation. Transformers and foundation models often utilize self-supervised learning (SSL), which, on the other hand, increases data efficiency by attaining guidance directly from data, rather than relying on expert knowledge through labels.^7–14^ In essence, SSL, widely used in general AI technologies, helps represent the next generation of AI in oncology, and many of its capabilities are yet to be discovered.

AI technologies can be divided in terms of scale into narrow and general AI, which differ in the sense that narrow AI is specialized for a single task, whereas general AI can perform multiple tasks. A promising type of general AI is foundation models, which are based on SSL and are able to digest substantial amounts of diverse data and are later adapted to perform very specific tasks. This ability of many foundation models to digest diverse datasets allows them to be multimodal, as they can process and utilize different types of information. Foundation models, which make predictions via data analysis, are considered a form of general predictive AI, whereas foundation models that generate new content, such as reports, fall under general Gen AI. Overall, a foundation model is defined as a large AI model that is trained on a great amount of often-unlabelled diverse data, allowing it to be adjusted for many different tasks.^4^^,^^7^^,^^15^ Foundation models have sparked discussion around the notion of artificial general intelligence (AGI). This hypothetical technology aims to perform a large range of tasks at a strength similar to the human intelligence.^16^ Building upon this same concept but specifically in medicine, generalist medical AI (GMAI) is an AI concept based on foundation models that can complete a great number of tasks with very little unlabelled data, and can generate recommendations, explanations, and annotations of images, showing advanced medical reasoning.^10^ Moreover, GMAI has a great number of applications in many medical areas, some of which will be discussed later in the article. Essentially, the concept of GMAI highlights that general AI models can solve difficult problems that were previously unsolvable, produce outputs from a diverse set of inputs, and represent medical knowledge, enabling the models to solve new tasks and explain results using accurate medical language.^10^^,^^17–19^

**Figure 2.**
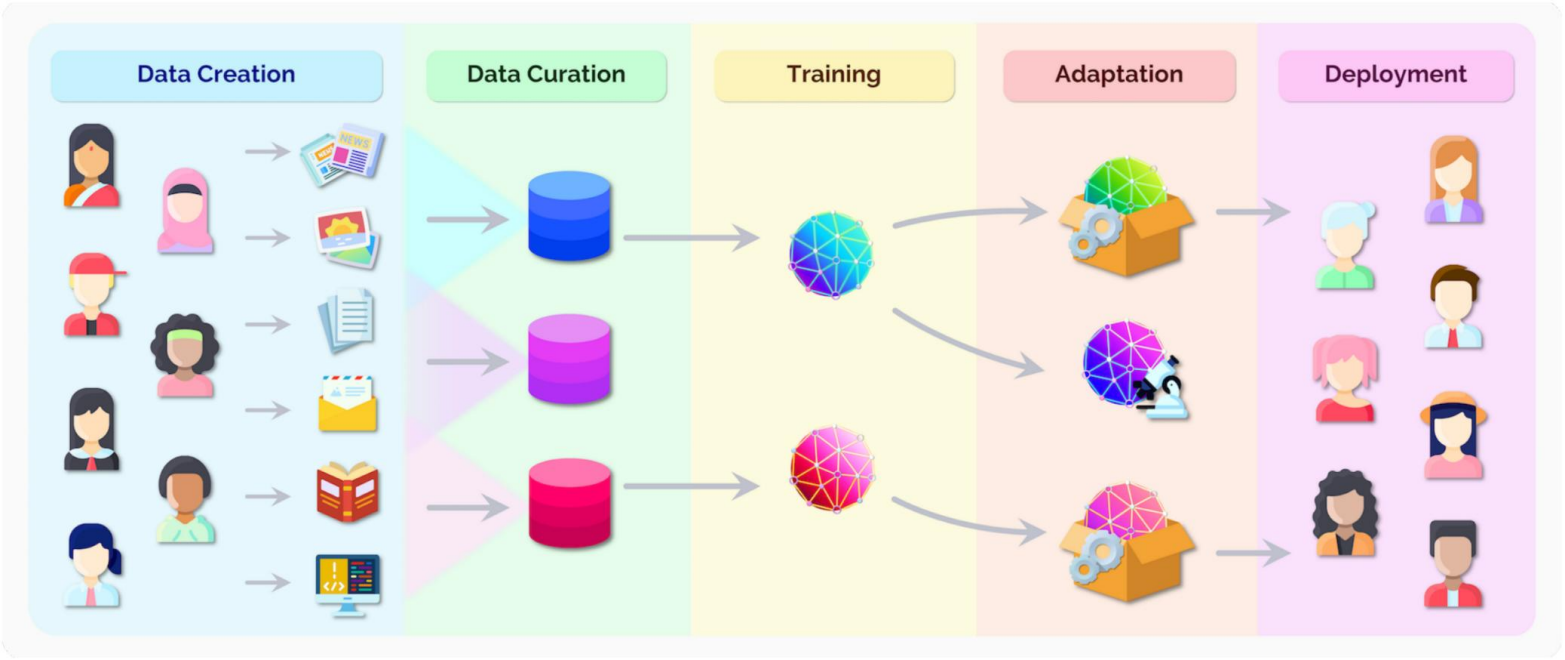
The development of foundation models. Foundation models undergo an extensive development process, beginning with the collection and creation of data to form datasets. These mainly unlabelled datasets are then utilized to train foundation models using self-supervised learning (SSL). After training, models can then be fine-tuned or adapted to perform specific tasks before being deployed for human use.^4^

Foundation models are developed in an extensive manner. The initial step is to gather data, which is then formed into a dataset, using vast amounts of information from the web or other data sources. In the case of foundation models, this information is usually unlabelled, allowing the models to identify relationships and patterns in the data without the need for human labelling.^4^ Once datasets are amassed, the model architecture is identified. Foundation models are often built upon transformers, which are adept for the large-scale identification of relationships and patterns in data. Next, training occurs; for foundation models, this is via SSL, where guidance is attained directly from the data without label use.^4^ After training, which often requires extensive computational power and time, comes evaluation and fine-tuning. Fine-tuning specifically requires task-specific data, which are typically labelled within a supervised learning framework. Models can be evaluated for a variety of downstream tasks, with their performances assessed. Models can then be adapted or fine-tuned for specific tasks, allowing for specialization in particular areas. Finally, models are then deployed for human use. Figure 2 provides an overview of the development of foundation models, highlighting data creation, data curation, training, adaptation, and deployment.^4^

Given the recent, promising innovations that have surrounded AI in medicine, there has been an increased desire to utilize such tools in clinical trials. Hence, steps have been taken to enhance AI validation in this area. For example, VeriSIM Life’s BIOiSIM platform utilizes AI and ML to replicate the effects of chemicals on whole bodily systems and individual organs.^20^ Furthermore, IQVIA has created a data-driven detection program that can be used in clinical trials, analysing patient symptoms and traits to recommend treatments, etc.^21^

**Figure 3.**
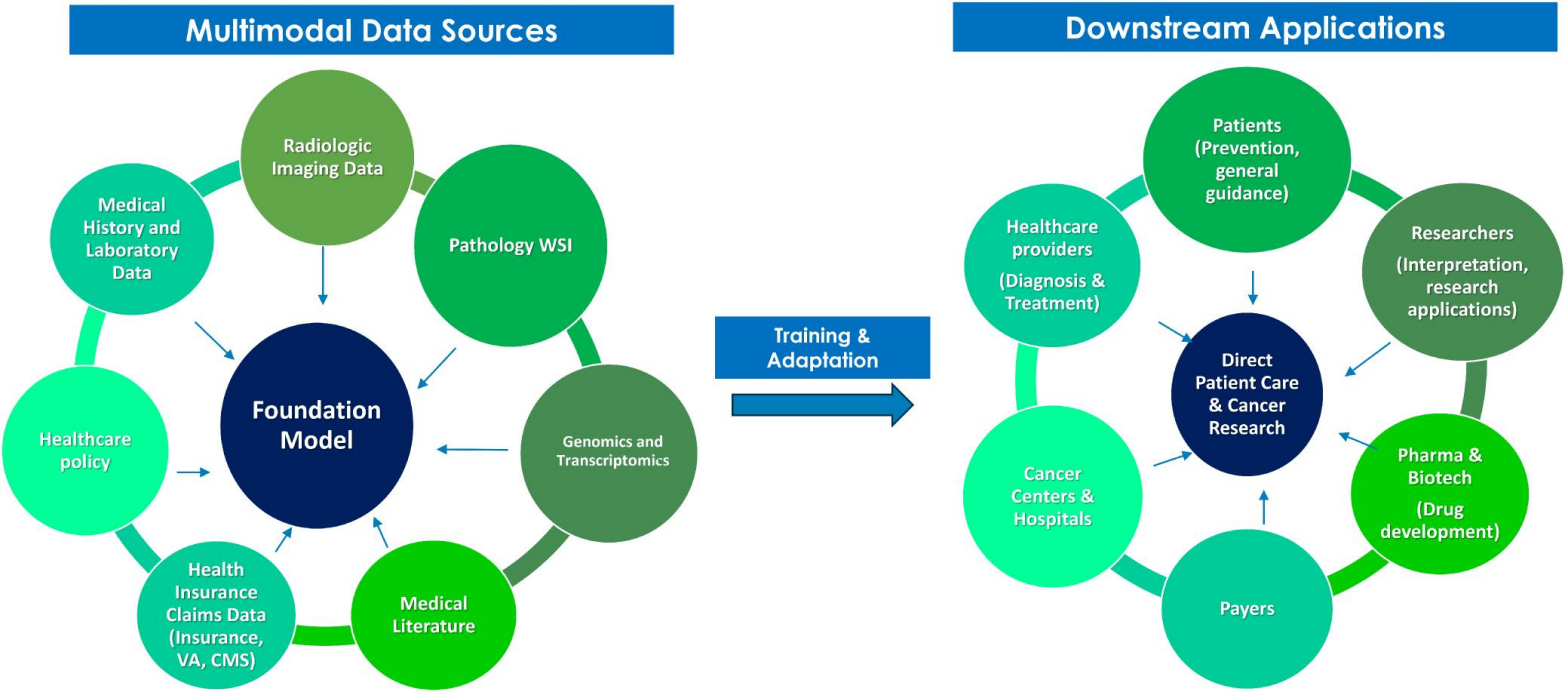
Foundation models in cancer diagnosis and treatment. Foundation models are envisioned to enable multiple applications in patient care and cancer research, resulting from endless feedforward loops of data sources and downstream applications. Abbreviations: CMS = Centers for Medicare and Medicaid Services; EMR = electronic medical record; VA = Veterans Affairs.

In this article, we will be reviewing growing AI applications, particularly of foundation models, in the diagnosis and treatment of cancer. This review article identifies, selects, and synthesizes information from a multitude of literature to provide a picture of the various applications of narrow and general AI in the diagnosis and treatment of cancer. To select examples from all applications, an extensive search was conducted on PubMed and arXiv databases to locate literature published mainly from 2018 onwards, focusing on the last 3 years for foundation models, that identifies current state-of-the-art regarding AI models, specifically foundation models, in the applications of radiology, pathology, precision medicine and cancer treatment, personalization of care through patient engagement, and surgical oncology. Attention is also paid to emerging forms of AI, such as AI agents. Table 1 summarizes each application area discussed in the article and its corresponding literature. Figure 3 is adapted from Bommasani et al and displays the different types of data that can be input into oncology foundation models, and the downstream tasks that can be performed resulting from these inputs.^4^

**Table 1.** Summary of sample foundation model applications for diagnosis and treatment of cancer.

AI application area	Key benefits	Representative examples	Reference
AI in radiology	General AI can allow foundation models to predict diagnosis and prognosis for a multitude of diseases, including cardiovascular and ocular disease, as well as cancer (see RETFound). Foundation model-segmented images spearhead increased precision in identifying the location and severity of abnormalities (see MedSAM). Generative foundation models can produce reliable reports for successful treatment planning and physician-patient communication (see RadFM).	*RETFound:* carries out ocular and cardiovascular disease incidence prediction and prognosis; performance can drop when tested on new cohorts with different demographics. *MedSAM:* specializes in segmenting radiological images; performance drops for less accounted-for modalities, and the model struggles with segmenting structures with vessel-like branching. *RadFM:* utilized to recognize modalities, diagnose diseases, generate reports, etc; however, there is a lack of 3D images in the model’s dataset, and missing metadata causes components such as imaging spacing to be missing.	^7^ ^,^ ^22^ ^,^ ^23^
AI in pathology	Predictive foundation models can subtype cancer using mostly unlabelled data, saving both money and time (see HIPT and Prov-GigaPath). Foundation model-driven digital whole-slide imaging analysis can lead to accurate treatment survival prediction beyond existing biomarkers, such as in immunotherapy (see HIPT). Foundation models can assist pathologists through the conduction of feature extraction and cellular/molecular analysis to separate cancerous tissue from healthy tissue.	*HIPT:* able to subtype cancer and predict survival, but can be limited by slides being excluded from its dataset due to the difficulty of patching and limited tissue content at 4096 × 4096 resolution. *Prov-GigaPath:* the model shows high-level performance in mutation prediction and cancer subtyping; however, cancer subtyping performance was much higher than mutation prediction. *MUSK:* and excels in image retrieval and classification and molecular biomarker prediction; requires substantial computing power as a multimodal model, which can have adverse environmental effects.	^24^ ^,^ ^25^ ^,^ ^26^
Precision medicine and cancer treatment	Predictive foundation models can analyse clinical patient data to weigh different treatment options and select personalized treatment plans based on predicted success. Generative foundation models can potentially suggest treatment procedures based on extracted information from unstructured medical text. Foundation models, using general AI, can predict onset for a wide range of diseases to ensure patients have ample time to prepare early and limit the risk of incidence (see Merlin).	*Med-Gemini:* can summarize medical visits, generate reports, answer clinical questions, and other tasks; that said, they require extensive validation *Med-PaLM M:* performs tasks including medical and visual question answering, report generation, and mammography and dermatology classification. That said, it can be limited by the lack of benchmarks for the development of this model, and there is also a lack of multimodal datasets available for the training of such models. *Merlin:* the model can perform phenotype classification, findings classification, cross-modal retrieval, report generation, and 5-year disease prediction; however, the model’s report generation is not yet optimized.	^27–29^
Personalization of cancer treatment through patient engagement	Generative foundation models such as LLMs can use empathy to have meaningful interactions with patients. Physicians can utilize generative foundation models to answer patient questions outside of appointment times, which can provide more direct help than the typical search engine (see Med-PaLM 2).	LLMs have been shown to express signs of empathy. General AI models could serve as personal chatbots to patients, but must be able to produce language that patients can comprehend while digesting wide varieties of patient information. *Med-PaLM 2:* can generate helpful and accurate responses to pressing patient questions; that said, there is a lack of understanding as to how LLM outputs compare to physician answers.	^10^ ^,^ ^30^ ^,^ ^31^
AI in surgical oncology	Predictive foundation model-driven tumour infiltration detection can improve success in complete tumour removal during surgery (see FastGlioma). Generative foundation models can potentially assist surgeons during procedures by answering questions based on live videos (see LlaVa-Surg).	*FastGlioma:* able to detect tumour infiltration during surgery. *LlaVa-Surg:* can answer open-ended questions pertaining to surgical videos and perform thorough evaluations on surgical video question-and-answer tasks; that said, outputs may struggle for universality.	^32^ ^,^ ^33^

Abbreviations: AI = artificial intelligence; LLMs = large language models.

## Narrow and general AI applications in radiology

Radiology is one of the main fields being affected by AI, as the fact that it can potentially perform radiology work is truly revolutionary. Using ML and deep learning, AI technology is able to detect pathologic lymph nodes just as a radiologist would.^34^ Consider QuantX, the first FDA-approved ML-powered system for assistance in the diagnosis of cancer in radiology, commercialized in 2017.^21^ The system can combine high-level image analysis with ML to help radiologists characterize and assess abnormalities and lesions in the breast, which could be cancerous. Ultimately, this could improve diagnostic accuracy, efficiency, and cost.^35^ QuantX can analyse MRI via kinetic colour maps, utilize and reference different data, and factor in QI scores for different lesions in order to assist in the accurate detection and diagnosis of breast cancer.^21^ This is highly impactful in clinical practice, as QuantX can enhance diagnosis by helping limit human errors.

**Figure 4.**
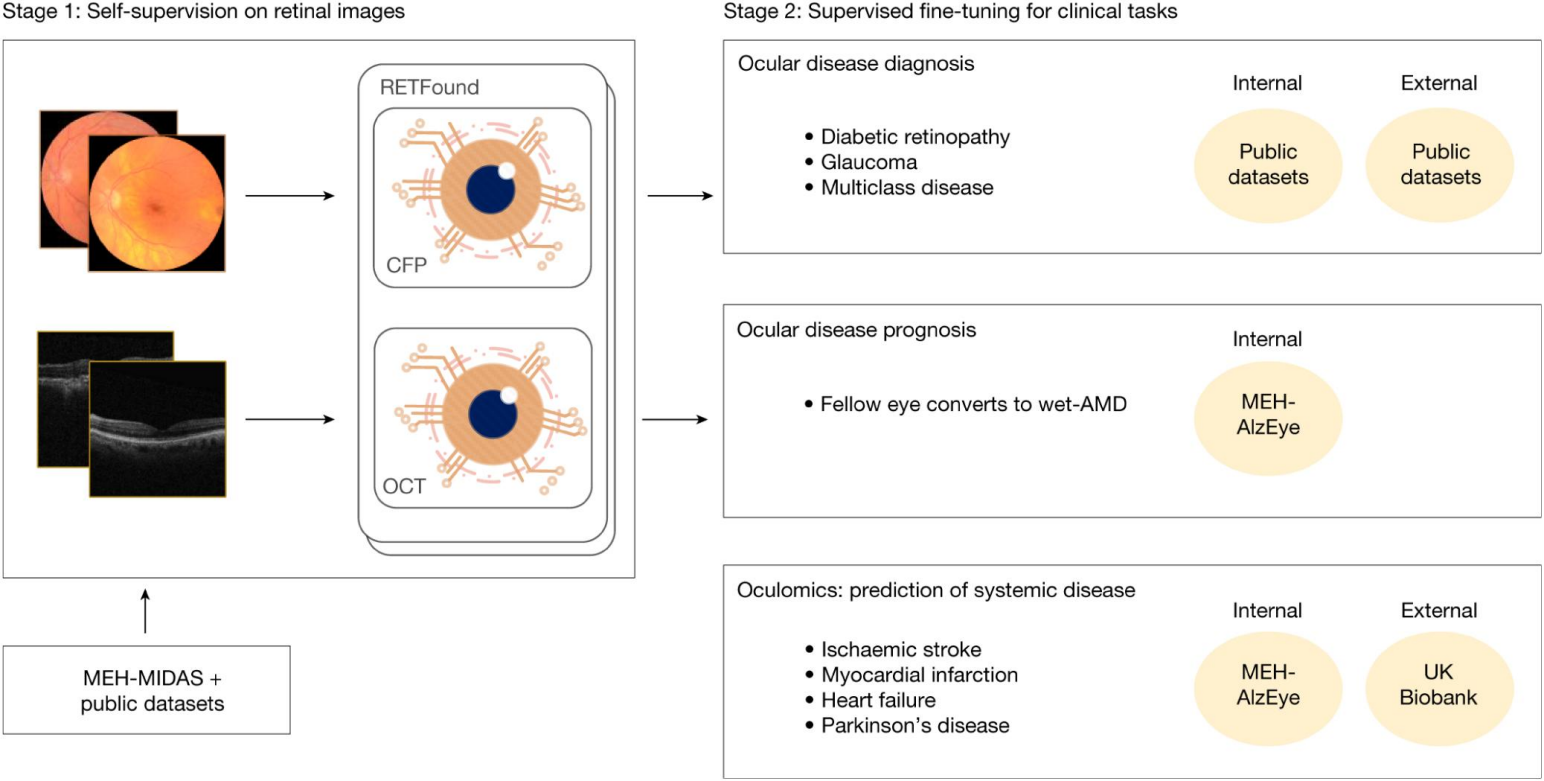
Graphic of the training and performance of foundation models (RETFound). Foundation models like RETFound utilize self-supervised learning as they are trained on numerous images (in this case, retinal images). The models are fine-tuned for various clinical tasks. RETFound, for instance, can be fine-tuned for ocular disease, systemic disease, and more.^7^

Focusing on the future, foundation models have already made their way into diagnostic radiology and have displayed massive potential. For instance, RETFound is a foundation model trained with numerous unlabelled retinal images obtained using leading methods of optical imaging, using them to enhance the detection of retina-related diseases. While QuantX is a narrow predictive AI technology, specialized for a single task, RETFound is an example of general predictive AI; while it still makes predictions based on data analysis, it can perform a wide range of specific tasks.^7^ The model shows high-level performance in diagnostics relating to multiple ocular, cardiovascular, and neurodegenerative diseases, with an area under the receiver operating curve (AUROC) of 0.862 for 1-year ocular disease prognosis and an AUROC of 0.737 for the prediction of myocardial infarction. Each score was significantly higher than the benchmark groups.^7^ Therefore, RETFound is a valuable assistant to medical professionals that can serve as a tool to enhance diagnostic accuracy and ensure that potential mishaps do not slip past physicians while streamlining many diagnostic processes that may consume significant amounts of time without AI involvement. This prospective success provides optimism for the future role of foundation models in improving clinical practice. However, when tested against new cohorts that have a different demographic profile, performance can significantly drop; for performance on ischaemic stroke, the AUROC of the adapted RETFound model dropped by 0.19 with optical coherence tomography and by 0.16 with colour fundus photography.^7^ That said, high label efficiency was also displayed, meaning that fewer labels were needed on average to get a task done. For instance, with regard to 3-year incidence prediction of myocardial infarction and heart failure, RETFound, with only 10% of training data, outperformed comparison groups using 45% and 50% of training data.^7^ This high performance with fewer labels can limit barriers to consistent use in clinics that may stem from issues with label scarcity, cost, and reliability, easing the transition of these foundation models into regular clinical utilization. Figure 4 by Zhou et al illustrates the development of RETFound models from pre-training to fine-tuning for various tasks.^7^ Another example of foundation models impacting radiology is MedSAM. MedSAM, mainly a general predictive AI technology, is a foundation model that specializes in segmenting medical images, a critical practice in radiology.^22^ The model is fine-tuned on a massive dataset spanning 10 imaging modalities and over 30 cancer types; the model showed promising results in a variety of tasks, such as polyp, where it displayed a Dice Similarity Coefficient (DSC) of 91.3% (IQR: 81.2-95.1%).^22^ However, the model’s performance on less accounted-for modalities such as mammography is hindered by an imbalance of representative image modalities in the training dataset. Furthermore, the model experiences difficulties in segmenting vessel-like branching structures due to possibly ambiguous bounding box prompts.^22^ These issues could be mitigated by fine-tuning the model to segment new tasks from complex structures such as vessels and less-represented modalities like mammography. Nonetheless, MedSAM’s high performance is greatly promising in radiology, as a foundation model for segmenting medical images can reduce the labour and time needed for segmentation, enhance consistency, and allow for the analysis of large-scale datasets.^22^ MedSAM can potentially improve accuracy and efficiency in radiological practice.

RadFM is another foundation model impacting clinical practice; RadFM, a form of general Gen AI for its ability to generate content and perform a wide array of tasks, is also trained on a massive dataset that covers 17 medical systems and over 5000 diseases.^23^ The model can be utilized to recognize modalities, diagnose diseases, generate reports, etc, and showed high-level performance on all tested tasks. For instance, on modality recognition, RadFM posted an accuracy score (ACC) of 92.95%, with the next highest score out of comparison groups of other foundation models being 84.25%.^23^ That said, limitations exist, such as a low number of 3D images in the model’s dataset. Moreover, there is metadata missing from the dataset’s 3D images, since these are downloaded from the internet. This can cause important components, such as imaging spacing to be missing, meaning that there is a lack of accurate distance measurement that could impede determinations such as tumour size.^23^ Even so, RadFM’s ability to analyse images at a high level provides great promise for its ability to impact radiology in a variety of ways, not only improving diagnostic accuracy and efficiency, but also aiding in generating excellent reports, answering questions, etc.^23^ Overall, the versatility and precision of Gen AI and general AI with foundation models warrants great optimism for the future of diagnostic radiology as SSL continues to improve and advance.

## Narrow and general AI applications in pathology

Pathology is another field where AI is making a substantial impact. The use of deep learning on digital pathology slides can boost the speed of interpretation and accuracy while also addressing subjectivity when examining slides.^36^ In fact, in a study involving whole-slide imaging (WSI) of breast cancer, where the performance of different algorithms was compared with that of several pathologists, some algorithms fared better than the pathologists.^36^^,^^37^ Furthermore, a deep learning algorithm, exemplifying narrow predictive AI, was able to accurately locate and classify tumours and detect the pattern of specific mutations when examining lung cancer slides.^36^^,^^38^

Recently, foundation models have been looking to address limitations involving current narrow AI models. Many current models only use a small sample of image tiles from each slide, causing important information from the overall slide to be missed.^24^^,^^39^ Moreover, public pathology data are not always available or of high quality.^24^ Second, it is difficult to create a model that can successfully capture both the patterns within individual tiles as well as the patterns present throughout an entire slide.^24^^,^^25^^,^^40–43^ Third, the rare foundation models that have been created using real data from patients have not been made accessible to the public.^24^ In recent years, foundation models are being developed to hopefully address these hindrances. One such model is the Hierarchical Image Pyramid Transformer (HIPT). This general predictive foundation model is trained on 10,678 gigapixel WSIs, 408,218 4096 × 4096 images, and 104 million 256 × 256 images spanning 33 cancer types.^25^ The model specializes in cancer subtyping and survival prediction; for instance, In non-small cell lung cancer (NSCLC) subtyping, HIPT achieved a macro-averaged AUROC of 0.923 ± 0.020, with the next best score being 0.857 ± 0.059 from a weakly-supervised competitor.^25^ That said, HIPT can be limited in the sense some slides may be excluded from its dataset since there is difficulty patching and limited tissue content at 4096 × 4096 resolution.^25^

**Figure 5.**
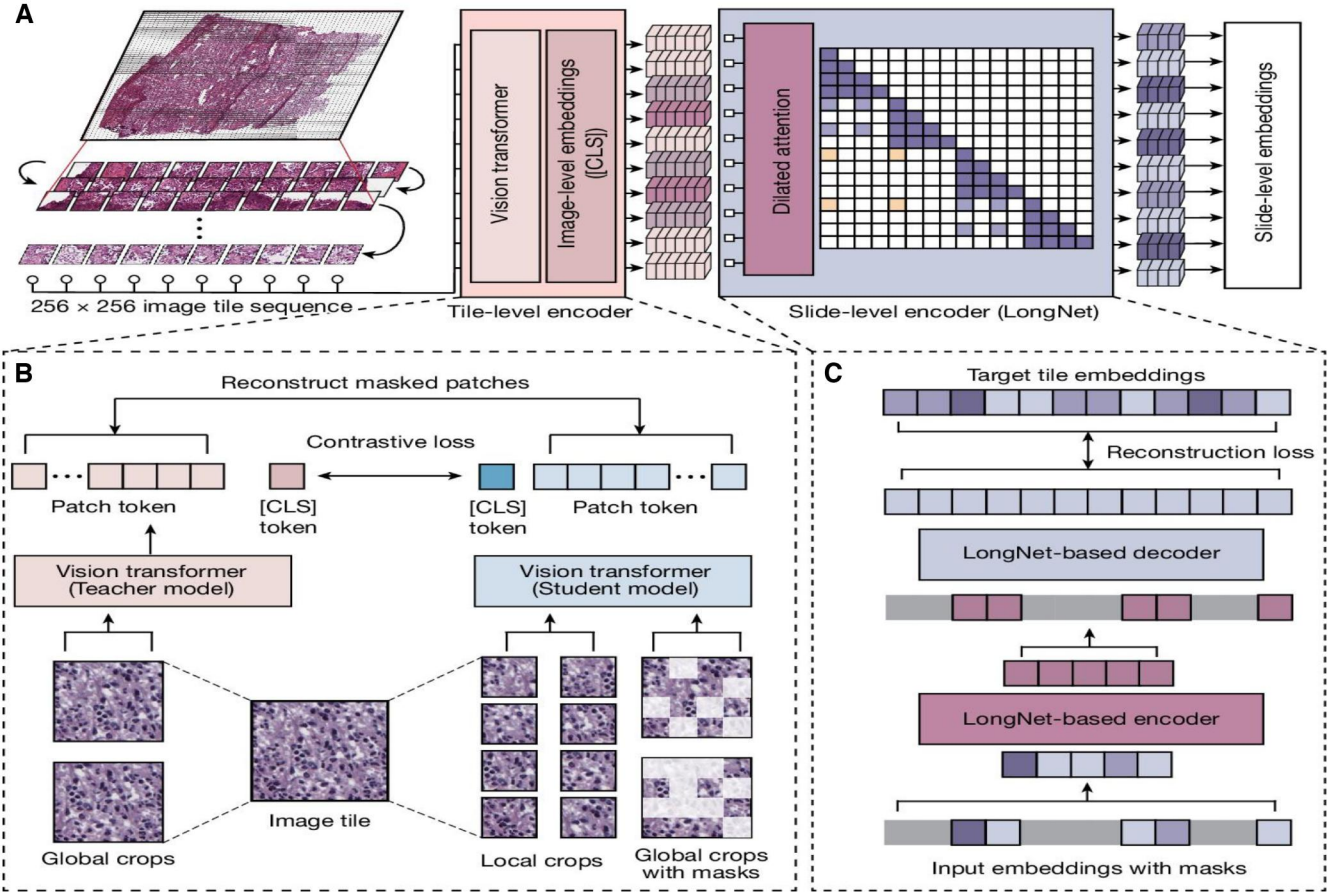
(A) An overview of Prov-GigaPath and foundation models in digital pathology. Prov-GigaPath and foundation models like it can divide each whole-slide image into many image tiles. A tile-level encoder then converts each image into a visual representation. Slide-level encoding then occurs, and the model enters pre-training. (B) Image tile-level pretraining is then carried out using a self-supervised framework. (C) Pretraining continues at the slide level utilizing a long-sequence modeling method. As pretraining concludes, finishing touches are applied to deem the model prepared for downstream applications.^24^

Another foundation model for pathological use, a general predictive AI technology, is Prov-GigaPath. This model is trained on 1.3 billion 256 × 256 pathology image tiles that cover 30,000 patients and 31 major tissue types.^24^ The model is pretrained on Prov-Path, a large set of digital pathology data, gathering valuable information from cancer centres.^24^ Pre-training is continued using GigaPath, a 2-stage transformer which helps represent image tiles as “visual tokens,” transforming a slide into a long succession of tokens.^3^^,^^24^ Prov-GigaPath shows outstanding performance in the subtyping of cancer and mutation prediction, providing promise for the role of foundation models in cancer detection and treatment.^25^ For instance, for the task of lung adenocarcinoma-specific 5-gene mutation prediction, Prov-GigaPath achieved an average AUROC of 0.708, which was significantly higher than competing methods consisting of other general-task models. Moreover, in cancer subtyping, the model’s average AUROC of 0.903 significantly outperformed leading competitors.^24^ Limitations, though, include that there is noteworthy variance between the model’s performance in mutation prediction vs cancer subtyping, with the latter being much higher. Although it still outperformed competitors in mutation prediction, other modalities could be used to enhance this aspect of Prov-GigaPath’s capabilities, since pathology image data may not be sufficient for the detection of some mutations.^25^  Figure 5 by Xu et al provides an overview of Prov-GigaPath, showing the model’s architecture and how it works with WSI.^24^

Another example of foundation models in pathology is MUSK. This is a general predictive vision-language foundation model that combines computer vision and NLP capabilities to understand and process both images and text.^26^ The model is pretrained on 50 million pathology images and 1 billion pathology-related text tokens. Then, it is pretrained on 1 million image-text pairs from the QUILT-1M model. The model is able to perform several pathological tasks such as image classification and retrieval and molecular biomarker prediction.^26^ In molecular biomarker prediction, MUSK posted an AUROC of 0.826 (95% CI, 0.813-0.839), with the next highest score being 0.786. Furthermore, in image retrieval and classification, for zero-shot image classification, where the model must classify images without the need for further training, MUSK outperformed the second-best models by 10.5%, 27.5%, 7.3%, and 10.1% when evaluated on 4 different datasets (a haematoxylin and eosin-stained pathology dataset, a skin cancer dataset, a dataset of histopathology scanning of lymph node sections, and a nuclei dataset of 19 different tissue types) and needing to distinguish multiple classes.^26^ This promising performance displays the potential of MUSK to improve diagnostic accuracy in pathology. However, as with any multimodal foundation model, the computational power required will be significant, especially due to the power needed to process different modes of data.^44^ That said, foundation models with SSL like HIPT, Prov-GigaPath, and MUSK provide a hopeful outlook for digital pathology in the future with regard to cancer detection, treatment, and more.

## Precision medicine and cancer treatment

Precision medicine can reap the benefits of AI as well.^45^ In fact, humans can use AI to analyse the case of a patient with cancer and predict which treatment method will work best, ensuring that the treatment received is the one that will provide the greatest outcomes.^34^ A massive part of the function of ML is to make predictions, which is why it can be so useful in precision medicine.^44^ For instance, its impact can be felt in the identification of causal genes, phenotypic and genetic heterogeneity, polygenic risk score, gene-gene interactions, and drug discovery.^44^ Not only that, but AI/ML can generate information to understand the cases of each specific patient to tailor ideal care plans to all individuals.^46^

Amazon Web Services (AWS) is an example of a commercial vendor that is taking advantage of AI in precision medicine. In fact, Amazon Comprehend Medical uses predictive AI technology such as ML and NLP to withdraw information from unstructured medical text such as radiology reports, clinical trial reports, and doctors’ notes.^46^ In this way, the technology can analyse diagnoses, procedures, prescriptions, etc to assist healthcare providers in the personalization of treatment, the identification of errors, and the improvement of safety.^46^ In a study where Amazon Comprehend Medical was evaluated for its performance in entity extraction, the model posted an F-score of 87%, underscoring its promising ability to analyse medical text.^47^ Involving AI in cancer diagnosis and treatment is a massive step in the right direction, as technologies like Amazon Comprehend Medical can extract and compare clinical trial and radiology reports, identifying valuable relationships between information that can provide important context to clinicians.^46^

The next generation of precision medicine and cancer treatment heavily involves foundation models with general Gen AI. A strong example of this is Med-Gemini, a family of self-trained models built on Google Gemini AI. These models can impact precision medicine in conducting certain tasks like summarization of medical visits, report generation, and clinical question answering (via the comprehension of medical datasets) that can enhance patient care. Med-Gemini is a prime example of the potential of SSL in medicine.^10^^,^^27^^,^^48–50^

Med-PaLM M is another example of how foundation models can impact precision medicine with general Gen AI. This foundation model interprets and processes a vast range of multimodal data, such as genomics, radiology reports, medical knowledge, dermatology, etc to perform various tasks including medical and visual question answering, report generation, and mammography and dermatology classification which can provide physicians with valuable information that can help tailor treatment plans to individual patients and optimize treatment success.^28^ That said, Med-Palm M is limited by the lack of benchmarks for the development of generalist biomedical AI like this model, which performs a diverse range of medical tasks. While specific benchmarks exist, there have not been many efforts made to unify these. There is also a lack of multimodal datasets available for the training of such models.^28^

Merlin is another general Gen AI model that also displays the application of foundation models in precision medicine. This vision-language foundation model is trained on millions of images, diagnosis codes, and tokens from CT scans, the electronic health record (EHR), and radiology reports and can perform tasks such as phenotype classification, findings classification, cross-modal retrieval, report generation and 5-year disease prediction, which can aid oncologists in precision medicine.^29^ Limitations exist, however, with Merlin’s report generation not yet being maximized for performance, as there are several parameters, specifically concerning the model’s adapter and LLM, that can be tuned to possibly enhance its abilities. That said, Merlin’s disease prediction ability is particularly noteworthy, as the model utilizes patient information to predict whether currently healthy patients will develop diseases such as chronic kidney disease, diabetes, cardiovascular disease, and others.^29^ In fact, with only 10% of labels used, for prediction of disease onset within 5 years, Merlin displayed an AUROC of 0.708 (95% CI, 0.692-0.723), outperforming an ImageNet pretrained model only for images by 4.4%.^29^ If Merlin finds a certain patient to be particularly susceptible to a disease, doctors can use this information to detect risks early and develop tailored treatment plans based on patient history and susceptibility.

## Personalization of cancer treatment through patient engagement

Patient engagement is important in the success of their treatment. Gen AI can improve patient engagement by further personalizing care and treatment plans.^34^ For instance, generative foundation models can be used for question-and-answer sessions with patients and can provide patients with support during decision-making. LLMs, for example, have been shown to express signs of empathy. Empathy can increase adherence to treatment and patient satisfaction while allowing medical professionals to understand the emotional states of patients.^30^ Moreover, with the concept of GMAI, general AI models can potentially provide patients with bedside support in decision-making, and they can give patients recommendations for care plans in the future.^10^ They can also possibly warn patients of imminent medical problems. In addition, general AI models can serve as a personal chatbot for patients. Models can power apps that can provide patients with insightful advice and explanations both inside and outside a clinical setting.^10^ However, for general AI models to act as chatbots, they must overcome 2 limitations. First, models must be able to use understandable and clear language that patients can comprehend. Second, models must be able to work with a wide range of information that is provided by patients, as some patient-provided data may be different than typical clinical data.^10^

Google-developed general generative model Med-PaLM 2 has shown promise for enhancing physician-patient interactions.^31^ Med-PaLM 2 is an LLM that is specifically trained on medical data and can simplify complex medical information into forms that are not only accurate, but also accessible to patients.^51^ Patients often have many inquiries regarding their medical situations, and sometimes do not have sufficient time to ask all these questions to their physicians. Instead of turning to a typical search engine, which can dump out information in an overwhelming manner, patients can turn to models like Med-PaLM 2 that are trained on massive amounts of medical data and can generate helpful and accurate responses to patient questions.^51^ However, there is a lack of understanding as to how LLM outputs compare to physician answers, and guidelines for best evaluating LLM performance by humans is an area needing further exploration.^51^ Despite this, by implementing interactive AI models like Med-PaLM 2 into patient care, patients can experience enhanced engagement with their treatment, as they will always have a reliable place to turn to when a question comes up. This ensures that patients are satisfied and involved in their treatment, improving treatment success.

## Narrow and general AI applications in surgical oncology

AI is currently being utilized in robotic surgical procedures. A commonly used form of robotic surgery is the da Vinci surgical system. This involves a surgeon controlling a robot through a console, with the surgeon essentially performing the surgery through the console and transmitting signals to robotic arms that perform the intended actions. In the da Vinci surgical system, the robot is not autonomous, meaning that it cannot act on its own without an operator controlling it.^52^ While this robotic surgical system lacks full automation, there have been steps taken towards incorporating automation into the da Vinci surgical system. For example, an algorithm was created that allowed for camera positioning on the robot to be autonomous. Furthermore, AI models have been created that guide surgeons through the suturing process.^53^ There are even certain breakthroughs that are starting to allow robots to suture themselves in very specific situations and circumstances.^52^^,^^54^ In addition, a deep learning model has been developed that can pinpoint areas that are safe for dissection by identifying surgical planes.^52^^,^^53^ These technologies, with the use of narrow predictive AI, have provided valuable and practical assistance to surgeons.

General AI with foundation models can be the motor behind effective AI assistants that can help surgeons and their teams with different procedures. For example, surgical foundation models can perform various tasks, such as annotating videos of surgeries and offering spoken feedback such as reading important information when surgeons face rare bodily cases and alerting surgeons when a procedural step is skipped.^10^^,^^55^ These models can also help with surgeries like endoscopic procedures that take place outside of the operating room. Moreover, foundation models can also reach conclusions during surgery about structures and situations that the model has not encountered before solely based on prior knowledge and experience.^10^

An example of a foundation model in surgery is FastGlioma. The general predictive model is pretrained on over 11,000 surgical specimens and 4 million distinct microscopy fields of view, and is able to detect tumour infiltration during surgery, allowing for maximal resection with optimal safety.^32^ In quantification and detection of tumour infiltration, FastGlioma displayed an average AUROC of 92.1 ± 0.9%, showing its ability to locate infiltrations at a high level.^32^ LlaVA-Surg is another foundation model that displays great potential in surgery. This multimodal LLM is trained on a dataset containing over 102,000 surgical video-instruction pairs from 2,201 surgical procedures.^33^ The model, which is general generative, is able to answer open-ended questions pertaining to surgical videos and can perform thorough evaluations on zero-shot surgical video question-and-answer tasks, displaying promise as a valuable assistant to surgeons.^33^ On zero-shot surgical question-and-answering tasks, LlaVA-Surg displayed a score of 2.45 (out of 5), with the next highest score out of the compared models being 1.32, which underscores the promise of the model’s performance.^33^ Despite this promise, LlaVA-Surg displays noteworthy limitations. For instance, the model is capable of “hallucination,” where it may produce a confident but incorrect recommendation or response.^33^ In addition, given that the model’s data source includes many rare cases, LlaVA-Surg’s outputs may struggle for universality and may not apply to wide-ranging clinical situations.^33^ Overall, foundation models are impacting surgical oncology in a variety of ways and show great promise for their potential use as important and helpful surgical assistants.

## Discussion

It is undeniable that AI warrants substantial promise for its ability to potentially revolutionize oncological practice. However, the technology does have limitations and hindrances that it must overcome to reach its full potential. For instance, defective AI algorithms can cause serious harm to patients via subsequent flawed treatment, the spread of misinformation, and more. Also, it is difficult to determine how some algorithms produce outputs, which raises the question of whether these nontransparent algorithms should be used in healthcare. There is also a risk that AI algorithms could be hacked, which could cause major harm to many patients regarding information security and privacy.^36^^,^^56–59^

Furthermore, the ethical concerns stemming from AI’s usage in medicine can have negative societal impacts, such as issues related to accountability for errors. Clinicians may over-rely on AI algorithms in patient care, placing excessive trust in AI recommendations that may lead to developed biases, spreading incorrect information, etc, that can go unchecked by physicians.^60^ Over-reliance can also lead to “deskilling,” where physicians may no longer be able to adequately check AI-based recommendations for mistakes, which can also lead to the spread of harmful information. This can have detrimental societal impacts, as patient health can be put at risk if AI-influenced decisions lead to mishaps in treatment or harmful patient decisions, decreasing treatment effectiveness and putting patients at risk for harm.^60^ Additionally, physicians who show compassion and emotion with patients often display greater treatment success; it is unlikely that AI is able to replicate such human-to-human interactions. Patients inherently prefer to be treated by humans, and AI-facilitated treatment can struggle to produce similar emotional interactions, potentially lowering treatment success and causing detriment to patients, negatively affecting society. Therefore, it is important that a human physician is heavily involved in all patient-related decisions while ensuring that AI-driven decisions are constantly checked for error.^60^

Despite the promise of AI, its software development also comes at a price. Obtaining AI software can be very expensive. Moreover, the use of AI can also have major environmental impacts. Demands for AI computer processing are causing dangerous increases in air pollution stemming from power plants and other generators that supply computer processing centres with electricity. For instance, backup generators in regional areas can lead to 10-fold increases in public health costs if electricity is emitted at the generators’ maximum level.^61^^,^^62^

Focusing on foundation models, since they are so versatile, they can be difficult to validate generally. In essence, it can be challenging to ensure that the work of foundation models is correct unless a specific task is defined and the foundation model is tuned for it.^63^ Moreover, the sheer size of foundation models can raise questions on potential consequences that may stem from their use. For instance, foundation models often contain billions of parameters, significantly more than the typical convolutional neural network (CNN), which may have thousands or millions. While foundation models use higher levels of unlabelled data than other AI technologies, potentially saving costs that may come from the use of expensive labels, other issues may rise from the size of foundation models. The massive number of parameters in a foundation model means that more graphics processing units (GPUs) are needed to be trained due to increased computational demands, etc, requiring larger amounts of energy.^64^ Additionally, LLMs, despite their great capabilities, can produce outputs that are either false or unsupported.^65^ This unreliability poses major risk, especially in medicine, where faulty radiology or pathology reports may negatively impact patient care. That said, entropy-based estimators for uncertainty are being developed, utilizing probability and quantifying when an LLM is likely to produce unreliable information in an effort to limit the impact of these “hallucinations.”^65^ There is promise that these methods can increase the range of applications where LLMs could be safely used.^65^

As discussed with the concept of GMAI, general AI models can provide many benefits to oncologists. However, their large-scale models can introduce biases that pose a serious risk. During training, datasets can underrepresent certain patient groups or show harmful statistical relationships, developing biases that are especially concerning given the size of these general AI models.^10^ To address this, models must be extensively validated to ensure that all patient groups are adequately represented, and consistent inspections must be performed on these models even after development to address issues that arise as general AI models come across new information and circumstances.^10^

Data privacy is also a concern with general AI models; they will have access to vast amounts of sensitive patient data during training, and due to their size, models may be susceptible to memorizing and repeating this training data to patients, potentially exposing confidential information.^10^ In order to mitigate this risk, personal information can be removed from medical documents when training general AI models, and the amount of data collected from each patient can be reduced.^10^ Although these limitations to all forms of AI may seem worrisome, AI is still at the helm of future innovations that can revolutionize society.

A recently emergent and promising AI technology is the AI agent. AI agents are autonomous programs that can interact with their environment, perform various tasks, and make decisions with AI. What differentiates AI agents from other forms of AI is that AI agents can act autonomously. On the other hand, other AI models require consistent human intervention to trigger an action based on model output. AI agents use a blend of ML, decision-making processes, and high-level algorithms in order to function efficiently, and their ability to carry out assignments without human involvement warrants a great amount of optimism, especially in healthcare. There are many types of AI agents, which can improve efficiency, enhance security, and much more. One example of an AI agent is Inner Monologue, which uses human feedback to recognize user preferences or comprehend unclear requests using context clues.^66^ Importantly, this technology does not require human initiation, which is what makes it an AI agent. Regarding medicine, AI agents can complete rudimentary tasks, assist in the diagnosis and treatment of diseases such as cancer, and analyse various modes of medical data. Additionally, single-agent systems utilize only a single AI agent, while multi-agent systems consist of multiple agents which collaborate to perform a task. AI agents often utilize the capabilities of foundation models, leveraging technologies such as LLMs to comprehend, reason, and act on information. In addition, the utilization of foundation models by agents allows them to break complex tasks into simpler ones. In essence, AI agents combine the abilities of foundation models with the agentic capability to interact with the environment and reason in an autonomous matter, resulting in an innovative technology that carries great promise.

The development of agentic AI further expands on the development of foundation models. AI agents combine foundation models (largely LLMs), computational frameworks, and field-specific tools to effectively perform scientific tasks.^67^ Agents are then trained on datasets that aim to assess the ability of the technology to plan, reason, and collaborate on tasks such as generating hypotheses, planning experiments, and analysing literature. LAB-bench is an example of a dataset used for planning and the evaluation of reasoning in biological research.^67^ Then, depending on the field or task, metrics are utilized to evaluate the performance of AI agents. These metrics can span from recall and prediction error for the tasks of scientific discovery and experimental prediction, to response coherence and task completion rates for planning and reasoning.^67^

AI agents are already making an impact in the medical field. In fact, agentic AI can be used to assist scientists with making new discoveries, which can help mitigate many of the current limitations of AI that may prevent the widespread clinical adoption of the technology. An AI co-scientist has been developed with the intent to discover original knowledge and create new research hypotheses.^68^ This technology consists of a multi-agent architecture with an unsynchronized task execution framework allowing for flexible compute scaling. The AI co-scientist is capable of repurposing drugs, discovering new targets, and explaining the mechanisms of anti-microbial resistance and bacterial evolution.^68^ The development of such technologies can spearhead discoveries and allow scientists to address the limitations of AI efficiently and accurately.

**Figure 6.**
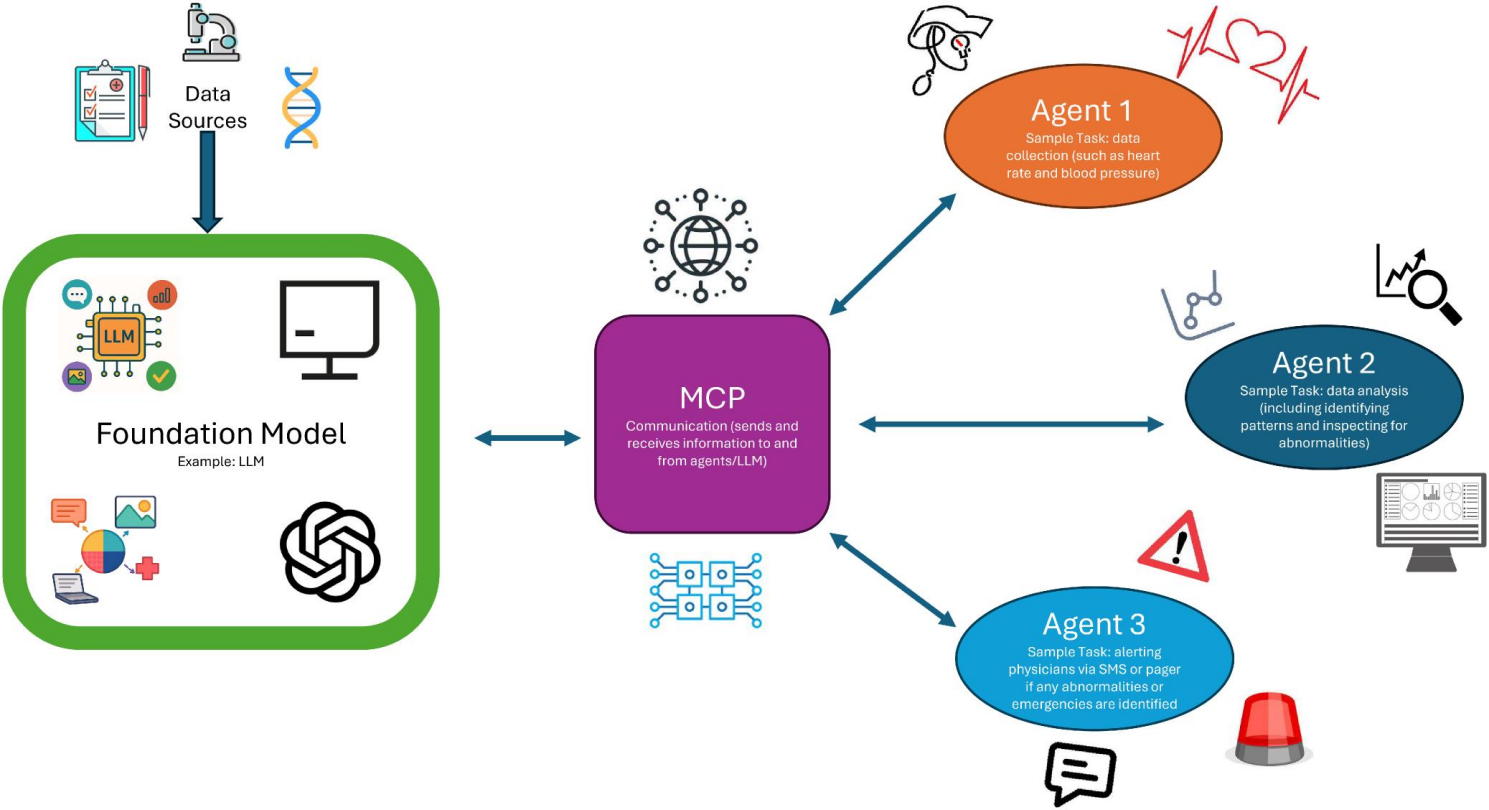
An example of the interactions of foundation models and a multi-agent system with MCP for medical applications. In a multi-agent system, MCP can spearhead efficient communication and collaboration between agents and foundation models by providing each technology with the information needed to carry out its tasks. In medicine, such a system can be used for patient monitoring, where individual agents can be responsible for gathering data, analysing this data, and alerting physicians of emergencies while being supervised and connected to a foundation model and each other via MCP. Abbreviation: MCP = model context protocol.

A newly released, promising technology is the model context protocol (MCP). This technology aims to standardize the way AI systems connect to data sources, looking to overcome challenges such as isolation from data.^69^ Regarding architecture, either AI applications, known as MCP clients, can be built that connect to MCP servers, or developers can expose their data through these servers.^69^ Essentially, LLM applications act as hosts to initiate connections, clients ensure that connections with servers within the host are maintained, and servers provide clients with prompts, tools, and context.^69^ Through the MCP, agentic AI models can access the data needed to effectively carry out tasks, helping to represent the future of AI in cancer research. Figure 6 depicts how MCP, foundation models, and AI agents can interact to facilitate medical tasks in a multi-agent system.

Although AI has many challenges to overcome before routine clinical use, its recent progress and displayed promise means that its role in clinical trials is continuously expanding. Training and validation on multi-institutional datasets, such as the ones available to cooperative groups including the Alliance for Clinical Trials in Oncology, ECOG-ACRIN, and the European Organization for Research and Treatment of Cancer (EORTC), have gained traction and interest. As discussed in the introduction, VeriSIM Life’s BIOiSIM platform, as well as IQVIA’s data-driven detection program, are examples of how AI validation in clinical trials is improving. That said, AI validation that meets regulatory requirements is still rather difficult, requiring extensive testing, explanation of algorithms, and inspection.^20^ There is also a lack of benchmark data currently that can validate AI-developed drugs in clinical trials, posing as a main obstacle to regular clinical use.^20^

Despite the promise of AI in precision oncology, barriers exist to its widespread clinical adoption. For example, regulatory obstacles such as a deficiency in established case laws regarding medical AI, physician concerns about legal responsibility for AI-made diagnostic errors, and potential issues with patient safety can cause hesitation for healthcare institutions. These hurdles make it difficult to navigate medical liability and to ensure that patients are optimally safe.^70^ Additionally, physicians must be able to trust the evidence that algorithms are based upon, and will likely not adopt AI tools without working understanding of how AI-based recommendations are made.^70^ Therefore, physician training is needed for clinicians to build confidence with AI tools needed for comfortable and routine adoption. In order to overcome these obstacles and promote increased integration of AI tools into clinical workflows, steps such as creating modules to educate oncologists about AI interpretations and applications, and establishing legal and regulatory frameworks for AI tools must be undertaken.^70^ The abundance of AI applications in oncology warrants optimism, but there are certain limitations to address to ensure the routine use of these technologies throughout the field of precision medicine.

The future of AI in oncology carries great promise, especially with the recent developments of agentic AI and MCP. That said, agentic AI is still limited in the sense that agents often rely on foundation models such as LLMs, which can produce faulty errors and biases. Moreover, insufficiencies or gaps in datasets may lead to biased or poor agentic performance; the AI co-scientist, for example, does not have much access to negative experimental data, meaning that it may not be exposed to a balanced view regarding datasets.^68^ Despite this, agentic AI and MCP still show great promise in spearheading future innovations in the field of cancer research. In essence, new and innovative forms of AI, such as foundation models and AI agents, while limited, are making optimistic breakthroughs in a variety of oncological areas, as discussed in this review. Table 2 summarizes the risks, impact benefits, and function of AI from a holistic view.

**Table 2.** Summary of function, risks, and impact benefits of AI.

AI element	Summary	Reference
Function of AI	AI systems function by merging large data sets with smart, iterative algorithms. If equipped with feedback learning, AI can examine its performance and increase its knowledge every time data is processed. AI can learn and grow from features and patterns in the data that it analyses, increasing its capabilities as it consumes more data.	^1^ ^,^ ^8^
Risks of AI—Applying to all AI Discussed (Predictive and Generative)	Defective AI algorithms can harm patients through flawed treatment, the spread of misinformation, etc Some algorithms are nontransparent; (black boxes); it is difficult to determine how they produce outputs. There is a risk that AI algorithms can be hacked, which can raise privacy concerns. Increased disparity due to biased training data.	^36^ ^,^ ^56–62^
Risks of AI—Specific to General AI/Foundation Models	Foundation models can be hard to validate due to their versatile nature. Foundation models are highly expensive and require a very large number of GPUs to train. Increased GPU training can heighten carbon dioxide output, harming the environment. LLMs can produce false or unreliable outputs that may negatively impact patient care. General AI models can develop biases and expose private patient information.	^10^ ^,^ ^64^ ^,^ ^65^

Abbreviations: AI = artificial intelligence; LLMs = large language models.

## Conclusions

Overall, AI has great potential in treating cancer through many different ways. In diagnostic radiology, foundation models have built off of past technologies to perform radiological tasks such as report generation, abnormality identification, and cancer detection, and at an enhanced accuracy. The use of emerging forms of AI in diagnostic imaging could possibly help locate bodily damage and discrepancies better and more efficiently than humans in the years to come. Moving on, pathology is yet another field impacted by AI, with foundation models looking to improve upon past methods in subtyping cancer, detecting diseases, and predicting mutations and biomarkers. Furthermore, AI can be of great use in precision medicine by analysing the medical situations of patients and predicting which treatment plan is best for each person. Extracting information from medical data via AI can further tailor treatment plans to each specific patient. Moreover, by improving personalization in treatment through patient engagement, AI can make plans easier to follow, meaning that more patients receive more successful treatment. Finally, the role of AI in surgical oncology was discussed. Successful robotics has been used with AI to carry out procedures, and SSL has more recently been applied to serve as a reliable surgical assistant for identification and question-answering tasks. With so many different current and future applications for AI in healthcare, it is difficult to deny the transformative potential that AI possesses in the field of medicine. That said, ethical concerns have surrounded AI, and there has also been some worry surrounding patient privacy, fairness, and legal responsibility. These concerns demand strategies and regulations to limit bias and ethical concerns. Nonetheless, as long as it is used responsibly, AI technology can be a highly valuable resource in medicine for years to come.
